# 单孔与三孔胸腔镜肺部手术后急慢性疼痛的对比研究

**DOI:** 10.3779/j.issn.1009-3419.2018.04.09

**Published:** 2018-04-20

**Authors:** 彩伟 李, 美青 徐, 广文 徐, 燃 熊, 汉然 吴, 明然 解

**Affiliations:** 230001 合肥，中国科学技术大学附属第一医院安徽省立医院胸外科 Department of Thoracic Surgery, The First Affiliated Hospital of USTC (Anhui Provincial Hospital), Hefei 230001, China

**Keywords:** 肺肿瘤, 胸腔镜手术, 术后疼痛, 疗效, Lung neoplasms, Video-assisted thoracic surgery, Post-operative pain, Outcomes

## Abstract

**背景与目的:**

通过比较分析单孔与三孔胸腔镜肺癌手术后急性和慢性疼痛的发生情况，探讨单孔胸腔镜手术能否进一步缓解肺癌患者术后急慢性疼痛的发生。

**方法:**

回顾性分析2016年1月1日-2017年6月30日在我院接受单孔或三孔胸腔镜下非小细胞肺癌手术的患者232例，其中131例接受单孔胸腔镜手术，101例接受三孔胸腔镜手术。比较分析两组患者临床和手术资料，以数字评定量表(numeric rating scale, NRS)评估两组患者术后第1、2、3、7、14天，3个月和6个月疼痛发生情况。

**结果:**

两组患者的一般临床资料无差异，均无围手术期死亡病例。单孔组在术后第1、2、7、14天NRS评分和术后第3、6个月慢性疼痛发生率方面较三孔组具有优势，差异具有显著性(*P* < 0.05)，而在手术时间、术中出血量、术后住院时间、胸管留置时间、术后第3天NRS评分方面差异无统计学意义(*P* > 0.05)。进一步对术后慢性疼痛进行单因素和多因素分析，结果显示手术时间、手术方式和术后第14天NRS评分是术后3个月、6个月发生慢性疼痛的危险因素(*P* < 0.05)。

**结论:**

单孔胸腔镜肺癌手术在非小细胞肺癌患者术后急性和慢性疼痛发生方面较三孔胸腔镜手术存在优势，手术时间缩短可减少术后慢性疼痛的发生，术后第14天疼痛发生是术后慢性疼痛的危险因素。

## Abstract (en)

**Background and objective:**

Through the comparative analysis of the acute and chronic pain postoperative between the single port and triple port video-assisted thoracic surgery to seek the better method which can reduce the incidence of acute and chronic pain in patients with lung cancer.

**Methods:**

Data of 232 patients who underwent single port -VATS (*n*=131) or triple port VATS (*n*=101) for non-small cell lung cancer (NSCLC) on January 1, 2016 to June 30, 2017 in our hospital were analyzed.The clinical and operative data were assessed, numeric rating scale (NRS) was used to evaluate the mean pain score on the 1^th^, 2^th^, 3^th^, 7^th^, 14^th^ days, 3^th^ months and 6^th^ months postoperative.

**Results:**

Both groups were similar in clinical characteristics, there were no perioperative death in two groups.In the 1^th^, 2^th^, 7^th^, 14^th^ days and 3^th^, 6^th^ months postoperative, the NRS score of the single port group was superior, and the difference was significant compared with the triple port (*P* < 0.05).There was no statistically significant difference between the two groups in operative time, blood loss, postoperative hospitalization time, duration of chest tube, the NRS scores in the 3 d (*P* > 0.05).Univariate and multivariate analysis of the occurrence on the chronic pain showed that the operation time, surgical procedure and the 14^th^ NRS score were risk factors for chronic pain (*P* < 0.05).

**Conclusion:**

The single port thoracoscopic surgery has an advantage in the incidence of acute and chronic pain in patients with non-small cell lung cancer.Shorter operative time can reduce the occurrence of chronic pain.The 14^th^ day NRS score is a risk factor for chronic pain postoperative.

肺癌的发病率和死亡率在全世界范围内一直居于所有恶性肿瘤的首位，以手术为主的综合治疗是可切除非小细胞肺癌(non-small cell lung cancer, NSCLC)的主要治疗手段^[1, 2]^。近年来，随着医学科学的发展，胸腔镜手术已成为治疗大多数胸外科疾病的常规术式。目前在国内外大多数医学中心，常规的胸腔镜手术一般选择3个-4个操作孔完成手术操作，相对于传统开胸手术，明显减少了手术的创伤^[3]^。自2011年Gonzalez等^[4]^首次报道了单孔VATS肺叶切除术以来，该术式在全世界范围内逐渐被广泛推广。单孔胸腔镜手术将切口减到一个，创伤更小、术后疼痛更轻、更符合美容要求，是近年来微创外科发展的主要方向之一^[5]^。

术后疼痛是胸部手术后最常见的症状之一，术后疼痛使患者恐惧咳嗽，增加了肺部并发症发生率，并对患者心理产生负面影响，严重影响患者术后康复过程。文献报道，相对于传统开胸手术，多孔胸腔镜手术能明显减少肺癌患者术后疼痛发生率^[6, 7]^。单孔胸腔镜手术将手术切口减少到最少，其是否可进一步减轻肺癌患者术后急慢性疼痛，相关研究较少。本研究收集中国科学技术大学附属第一医院安徽省立医院胸外科自2016年1月1日-2017年6月30日接受胸腔镜肺叶切除术的NSCLC患者232例，其中131例接受单孔胸腔镜手术，101例接受三孔胸腔镜手术。比较分析两组患者临床病例资料、围手术期资料和术后急慢性疼痛发生率等情况，现报告结果如下。

## 1 资料与方法

### 1.1 一般资料

回顾性分析2016年1月1日-2017年6月30日，在我院连续接受手术的NSCLC患者，共计531例。病例纳入标准：(1)病理证实为NSCLC；(2)手术方式为单孔或三孔胸腔镜肺叶切除手术，接受系统性肺门及纵隔淋巴结清扫；(3)能理解NRS，并知情同意的患者。剔除标准：(1)术前有慢性疼痛及服用阿片类等疼痛药物病史的患者；(2)具有精神症状患者；(3)术后病情危重或无法配合疼痛评估或随访；(4)术后6个月内疾病复发，慢性感染或再次手术的患者；(5)病例资料不完整。

基于以上标准，共232例患者纳入本研究。其中男性125例，女性107例；年龄17岁-84岁，中位年龄(61.21±10.51)岁。单孔胸腔镜组131例，三孔胸腔镜组101例。治疗前检查包括：胸部CT平扫+增强、支气管镜、头颅磁共振、全身骨扫描、心电图、肺功能，年龄 > 65岁患者加做超声心动图，术前化验为常规。肿瘤分期采用AJCC第8版TNM分期系统。

### 1.2 手术方式

单孔组：患者静脉吸入复合全麻，健侧卧位，单肺通气。取患侧腋前线和腋中线之间第4或第5肋间3.0 cm小切口，切口放置切口保护套。先置入胸腔镜探查胸腔有无粘连及播散结节，再确定病灶位置及肺门解剖情况。根据叶裂发育情况选择游离顺序，若肺裂发育良好优先处理动脉分支，若叶裂发育不佳，可采用" 隧道式" 或" 单向式" 方法切除。5 mm以下血管采用双重结扎后超声刀离断，5 mm以上血管、气管和发育不良的肺裂采用内镜下直线切割缝合器离断。右侧肺癌常规探查并清扫第2R、3A、3P、4R、7、8、9、10组淋巴结及肺内淋巴结，左侧肺癌常规探查并清扫第4L、5、6、7、8、9、10组淋巴结及肺内淋巴结。三孔组：麻醉方式同体位同单孔组，取腋前线第4或第5肋间3.0 cm切口为主操作孔，腋中线第7或第8肋间1.0 cm切口为观察孔，腋后线第7肋间1.5 cm切口为辅助操作孔。术中助手经辅助操作孔协助暴露术野，需要时可经辅助操作孔置入切割缝合器。肺叶切除及淋巴结清扫同单孔组。两组患者术后均采用舒芬太尼自控静脉镇痛泵，术后第2天均予以拔除。

### 1.3 随访及疗效观察指标

根据国际疼痛研究协会将术后急性疼痛定义为术后2周内的疼痛，慢性疼痛定义为术后疼痛持续超过3个月^[6]^。采用数字评定量表(numeric rating scale, NRS)评估患者术后急性疼痛的程度。0分表示无痛，10分表示剧烈疼痛。根据评估结果分为无痛(NRS=0)、轻度(NRS 1-3)、中度(NRS 4-6)和重度(NRS 7-10)疼痛4个等级。分别于术后1、2、3、7、14天，3个月，6个月分别观察并记录一次。采用门诊定期复诊和电话随访两种方式进行随访，观察术后第1、2、3、7和14天，3个月和6个月的疼痛情况，包括疼痛部位、NRS评分和疼痛性质。

### 1.4 统计学方法

应用SPSS 19.0统计学软件进行数据分析，计量资料以均数±标准差(Mean±SD)表示，组间比较采用独立样本*t*检验；计数资料以实际例数及所占百分比表示，两组率比较采用*χ*^2^检验，采用*Pearson*进行单因素相关性分析，应用*Logistic*回归分析慢性疼痛的影响因素，均以*P* < 0.05为差异有统计学意义。

## 2 结果

### 2.1 一般资料

共计随访患者232例，其中单孔组131例，三孔组101例。两组均无围手术期死亡患者。在年龄、性别、身高、体重、肿瘤位置和TNM分期方面比较，差异无统计学意义(*P* > 0.05)，两组患者临床资料具有可比性(表 1)。

**1:** 232例患者临床特征 Clinical characteristics of 232 patients

Variables	Single port (*n*=131)	Triple port (*n*=101)	*F*/*Z*/*χ*^2^	*P* value
Age (Mean±SD, yr)	60.85±10.46	61.68±10.60	0.36	0.549
Weight (kg)	67.37±10.11	67.81±10.62	0.11	0.745
Height (cm)	166.00±7.73	165.97±7.87	0.00	0.977
Gender			3.06	0.080
Male	64 (51.20%)	61 (48.80%)		
Female	67 (62.62%)	40 (37.38%)		
Location			3.41	0.492
Right upper lobe	53 (63.10%)	31 (36.90%)		
Right middle lobe	10 (62.50%)	6 (37.50%)		
Right lower lobe	18 (48.65%)	19 (51.35%)		
Left upper lobe	31 (54.39%)	26 (45.61%)		
Left lower lobe	19 (50.00%)	19 (50.00%)		
Pathological stage			0.42	0.810
Stage Ⅰ	74 (56.49%)	57 (43.51%)		
Stage Ⅱ	51 (55.43%)	41 (44.57%)		
Stage Ⅲ	6 (66.67%)	3 (33.33%)		

### 2.2 手术和术后疼痛随访情况

两组患者平均术后住院天数(8.14±4.917)天；平均胸管留置时间为(3.91±1.160)天；手术时间平均为(194.81±73.166)min；术中平均出血量为(101.90±37.136)mL。单孔胸腔镜组在术后第1、2、7、14天NRS评分和术后第3个月、6个月NRS评分方面，结果优于三孔胸腔镜组，差异具有显著性(*P* < 0.05)。单孔组与三孔组术中淋巴结平均清扫数目分别为(15.68±3.259)枚、(15.26±3.440)枚，无明显差异(*P* > 0.05)。在手术时间、术中出血量，术后住院时间，胸管留置时间，术后第3天NRS评分方面，单孔组与三孔组比较无明显优势(*P* > 0.05)(表 2)。

**2:** 比较2组术中情况和术后疼痛评分情况。 Comparison of the two groups with intraoperative statue and postoperative pain evaluation

Variables	Single port (*n*=131)	Triple port (*n*=101)	*F*/*Z*/*χ*^2^	*P* value
Operative time (min)	195.78±67.52	193.55±80.23	0.05	0.819
Blood loss (mL)	101.56±35.99	102.33±38.75	2.00	0.877
Hospital-stay postoperative (d)	8.03±4.97	8.26±4.86	0.15	0.694
Duration of chest tube (d)	3.81±1.11	4.02±1.22	1.93	0.166
Acute pain (NRS)				
1^th^ day	4.19±1.47	5.16±1.77	4.29	0.000
2^th^ day	3.55±1.76	4.04±2.02	4.06	0.045
3^th^ day	3.22±1.63	3.09±1.12	0.25	0.803
7^th^ day	2.25±1.53	2.66±1.47	4.28	0.039
14^th^ day	1.88±1.40	2.43±1.47	8.36	0.004
Chronic pain (NRS)				
3 month			19.59	0.000
No	97 (67.83%)	46 (32.17%)		
Yes	34 (38.20%)	55 (61.80%)		
6 month			13.02	0.000
No	113 (62.78%)	67 (37.22%)		
Yes	18 (34.62%)	34 (65.38%)		
NRS:numeric rating scale.				

### 2.3 术后影响慢性疼痛的单因素分析和多因素分析

分别对相关因素和术后3、6个月NRS评分进行相关分析，结果显示手术时间，术后第7、14天NRS评分和手术方式与术后3个月慢性疼痛发生率存在相关性(*P* < 0.05)。而在术后6个月，手术时间，术后第7、14天NRS评分，手术方式，肿瘤位置和肿瘤分期均显示存在相关性(*P* < 0.05)。为此，进一步对相关因素进行*Logistic*回归分析，结果显示手术时间、术后14天NRS评分和手术方式是术后慢性疼痛的主要危险因素(*P* < 0.05)(表 3、表 4)。

**3:** 术后影响慢性疼痛的相关因素分析 Single factor analysis of chronic pain after operation

Variables	Chronic pain (3 month)					Chronic pain (6 month)			
	No	Yes	*F*/*χ*^2^	*P* value		No	Yes	*F*/*χ*^2^	*P* value
Age	60.55±10.95	62.26±9.73	1.47	0.226		60.83±10.54	62.54±10.40	1.07	0.302
Weight	67.38±10.07	67.84±10.75	0.11	0.743		67.18±9.98	68.88±11.39	1.11	0.294
Height	166.36±7.78	165.39±7.77	0.84	0.359		165.78±7.67	166.69±8.16	0.55	0.458
Operative time	182.38±62.44	214.78±84.30	2.94	0.003		186.37±67.89	224.04±83.35	3.17	0.001
Blood loss	101.82±34.73	102.02±40.92	0.00	0.967		102.31±35.52	100.48±42.61	0.10	0.755
Duration of chest tube	3.97±1.18	3.81±1.13	1.08	0.299		3.95±1.13	3.77±1.25	0.98	0.323
1^th^ day NRS	4.49±1.62	4.82±1.76	2.14	0.144		4.53±1.67	4.90±1.68	1.97	0.161
2^th^ day NRS	3.78±1.79	3.75±2.05	0.01	0.927		3.76±1.87	3.81±1.96	0.03	0.861
3^th^ day NRS	3.16±1.53	3.18±1.25	0.27	0.788		3.16±1.44	3.19±1.39	0.02	0.889
7^th^ day NRS	2.10±1.49	2.95±1.41	18.64	0.000		2.16±1.46	3.38±1.30	29.95	0.000
14^th^ day NRS	1.72±1.29	2.75±1.49	31.33	0.000		1.89±1.35	2.88±1.54	20.29	0.000
Gender			0.31	0.579				0.10	0.756
Male	75 (60.00%)	50 (40.00%)				96 (76.80%)	29 (23.20%)		
Female	68 (63.55)	39 (36.45%)				84 (78.50%)	23 (21.50%)		
Surgery			19.59	0.000				13.02	0.000
Single port	97 (74.05%)	34 (25.95%)				113 (86.26%)	18 (13.74%)		
Triple port	46 (45.54%)	55 (54.46%)				67 (66.34%)	34 (33.66%)		
Tumor location			4.10	0.393				15.22	0.004
Right upper lobe	49 (58.33%)	35 (41.67%)				66 (78.57%)	18 (21.43%)		
Right middle lobe	11 (67.75%)	5 (31.25%)				14 (87.50%)	2 (12.50%)		
Right lower lobe	27 (72.97%)	10 (27.03%)				29 (78.38%)	8 (21.62%)		
Left upper lobe	36 (63.16%)	21 (36.84%)				50 (87.72%)	7 (12.28%)		
Left lower lobe	20 (52.63%)	18 (47.37%)				21 (55.26%)	17 (44.74%)		
Pathological stage			1.18	0.554				7.14	0.028
Stage Ⅰ	82 (62.60%)	49 (37.40%)				100 (76.34%)	31 (23.66%)		
Stage Ⅱ	57 (61.96%)	35 (38.04%)				76 (82.61%)	16 (17.39%)		
Stage Ⅲ	4 (44.44%)	5 (55.56%)				4 (44.44%)	5 (55.56%)		

**4:** 手术后患者慢性疼痛影响因素的*Logistic*回归分析 *Logistic* regression analysis of influencing factors of chronic pain postoperative

Variables	OR	OR (95%CI)	*P* value
Chronic pain (3 month)			
Operative time	1.006	(1.002-1.011)	0.001
14^th^ day NRS	1.634	(1.313-2.032)	
Surgery	3.179	(1.740-5.809)	0.000
Constant	0.009	(0.002-0.041)	0.000
Chronic pain (6 month)			
Operative time	1.006	(1.002-1.011)	0.003
14^th^ day NRS	1.532	(1.191-1.972)	0.001
Surgery	3.193	(1.536-6.639)	0.002
Tumor location			
Right upper lobe	1		
Right middle lobe	0.723	(0.137-3.810)	0.702
Right lower lobe	1.020	(0.352-2.951)	0.971
Left upper lobe	0.457	(0.161-1.296)	0.141
Left lower lobe	2.748	(1.081-6.986)	0.034
Pathological stage			
Stage Ⅰ	1		
Stage Ⅱ	0.672	(0.312-1.450)	0.312
Stage Ⅲ	2.041	(0.394-10.560)	0.395
Constant	0.004	(0.000-0.029)	0.000

## 3 讨论

胸部手术后疼痛是一种混合性疼痛，主要包括神经病理性疼痛和肌筋膜疼痛。手术引起的局部组织损伤导致感受器自发放电，可导致机体对外周刺激的敏感性增加，同时这种自发放电可导致中枢神经系统改变引起中枢痛觉敏化。术后疼痛导致患者咳嗽排痰受阻，进而增加了患者术后肺部并发症发生率。同时，术后疼痛对患者心理产生严重的负面影响，进一步影响了患者术后的病情恢复。因此，如何在围手术期运用各种手段缓解肺癌患者术后急慢性疼痛是胸外科医师的研究热点。单孔胸腔镜手术在不影响手术范围的前提下，将手术切口减少到1个，最大限度地减少了手术的创伤，已成为近年来胸腔镜手术发展的主要趋势之一。从理论上讲，单孔胸腔镜手术在缓解患者术后疼痛方面存在以下优势：(1)最大限度地减少了肋间神经的损伤，降低了患者术后切口疼痛；(2)使用软性切口保护套，避免了戳卡和副操作孔器械使用对切口的反复挤压和摩擦导致的术后切口疼痛；(3)腋后线副操作孔处神经肌肉丰富，对损伤而引起疼痛反应较为敏感。(4)更为美观的手术切口患者主观上更能接受，提高了患者术后的生活质量。本研究发现，相对于三孔胸腔镜手术，单孔胸腔镜手术在肺癌患者术后急性和慢性疼痛发生率方面均存在明显优势。进一步论证了该术式在缓解肺癌患者术后疼痛和快速康复方面的作用。

组织创伤所导致的外周敏化在术后急性疼痛中起主导作用。胸部手术后急性疼痛常常较为剧烈，特点为卧床休息时疼痛减轻，咳嗽时达到疼痛峰值，导致患者术后早期活动及排痰受阻，增加术后并发症及住院时间。Wang等^[8]^对比了开胸和三孔胸腔镜肺部手术后急性疼痛的发生率分别为61%和34%，胸腔镜手术在术后急性疼痛发生率方面具有明显优势。Ji等^[9]^对比研究163例接受单孔胸腔镜手术后第1、2天的中重度疼痛发生率分别为14.7%、11.0%，而同期272例接受三孔胸腔镜手术患者的发生率分别为24.6%、21.2%，单孔组具有明显优势。Hirai等^[10]^也得出单孔胸腔镜手术较三孔胸腔镜手术在术后急性疼痛方面具有明显优势。本研究结果显示在单孔组术后第1、2、3、7、14天的中重度疼痛发生率分别为56.48%、45.04%、40.46%、19.85%、12.98%，三孔组分别为69.30%、47.52%、43.56%、27.72%、23.76%，在术后第1、2、7和14天的疼痛评分方面，单孔组更具有优势。我们认为其主要原因在于，单孔手术最大限度的减少了肋间神经的损伤，另外，软性切口保护套的使用避免了戳卡和副操作孔器械使用对切口反复挤压和摩擦导致组织创伤而引起的外周敏感化有关。

国际疼痛研究协会将术后慢性疼痛定义为由手术引起，继发于术后急性疼痛且持续时间超过个3个月的疼痛，又称为术后慢性疼痛综合征。现在更多观点认为，发生于术后，伤口已完全愈合，但仍存在的疼痛均属于慢性疼痛的范畴^[6]^。近年来，越来越多的临床医务人员对术后慢性疼痛的治疗和预防提高了重视，但术后慢性疼痛的发生率仍然较高。术后慢性疼痛不仅严重影响患者的生活质量，同时对患者的心理产生严重创伤，导致患者怀疑肿瘤治疗的总体效果。Bendixen等^[11]^通过对比研究多孔胸腔镜(103例)与传统开胸(103例)手术后52周随访发现中重度疼痛的发生率分别为9.2%和13.6%，结果有统计学差异。Wildgaard等^[12]^通过研究分析了47例进行胸腔镜手术的患者，结果显示术后3个月11%的患者出现慢性疼痛，结果低于开胸手术相关文献报道结果(25%-50%)。本研究发现，单孔组患者术后3、6个月的疼痛发生率分别为25.95%、13.74%，三孔组分别为54.46%、37.67%，单孔组在慢性疼痛发生率方面存在明显优势。我们分析其主要原因在于单孔胸腔镜手术对患者肋间组织损伤更小，急性疼痛控制更好，且患者主观上更能接受更为微创的手术所致。

术后慢性疼痛发生机制非常复杂，文献报道在发生慢性疼痛的患者中大部分为神经病理性疼痛，还有相当一部分患者为心因性疼痛。Gottschalk等^[13]^报道在传统开胸手术中，出现急性疼痛较敏感以及住院期间疼痛缓解不明显的患者往往会演变为慢性疼痛。Wang等^[8]^研究结果显示术后5天出现活动后疼痛敏感和重度疼痛，是发生术后慢性疼痛的危险因素。据文献报道这可能与阿片类基因突变有关，此外中枢及周围的炎症反应系统可能也参与其发病机制^[13, 14]^。在本组病例中，术后发生慢性疼痛的患者中，52%曾出现急性疼痛。在相关性分析中发现，术后3个月和6个月疼痛与患者手术时间、术后第14天NRS评分和手术方式存在显著的相关性，接受单孔手术，手术时间短的患者和术后14天疼痛控制良好的患者发生慢性疼痛的发生率明显降低。因此，更小的手术创伤和更好的控制术后急性疼痛是减少患者术后慢性疼痛发生率的关键因素。

综上所述，相对于三孔胸腔镜手术，单孔胸腔镜可以进一步减轻肺癌患者术后的急性和慢性疼痛，加速患者术后的康复。术后14天出现术后疼痛是发生术后慢性疼痛的危险因素。本研究为回顾性对比研究，存在一定的病例选择性偏倚。其结果有待更大样本量和前瞻性随机对照研究进一步验证。

## References

[1] Yin HH, Tse MM, Wong FK (2012). Postoperative pain experience and barriers to pain management in Chinese adult patients undergoing thoracic surgery. J Clin Nurs.

[2] Li C, Ma HT, He JK (2013). Clinical analysis of thoracoscopic lobectomy in the treatment of peripheral lung cancer with single utility port. Zhongguo Fei Ai Za Zhi.

[3] Xie D, Chen C, Jiang GN (2016). Evolution and development trend of lung cancer surgical incision. Zhongguo Fei Ai Za Zhi.

[4] Gonzalez D, Paradela M, Garcia J (2011). Single-port video-assisted thoracoscopic lobectomy. Interact Cardiovasc Thorac Surg.

[5] Hao ZP, Cai YX, Fu SL (2016). Comparison study of post-operative pain and short-term quality of life between uniportal and three portal video-assisted thoracic surgery for radical lung cancer resection. Zhongguo Fei Ai Za Zhi.

[6] Bayman EO, Parekh KR, Keech J (2017). A prospective study of chronic pain after thoracic surgery. Anesthesiology.

[7] McElnay PJ, Molyneux M, Krishnadas R (2015). Pain and recovery are comparable after either uniportal or multiport video-assisted thoracoscopic lobectomy: an observation study. Eur J Cardiothorac Surg.

[8] Wang H, Li S, Liang N (2017). Postoperative pain experiences in Chinese adult patients after thoracotomy and video-assisted thoracic surgery. J Clin Nurs.

[9] Ji C, Xiang Y, Pagliarulo V (2017). A multi-center retrospective study of single-port versus multi-port video-assisted thoracoscopic lobectomy and anatomic segmentectomy. J Thorac Dis.

[10] Hirai K, Takeuchi S, Usuda J (2016). Single-incision thoracoscopic surgery and conventional video-assisted thoracoscopic surgery: a retrospective comparative study of perioperative clinical outcomesdagger. Eur J Cardiothorac Surg.

[11] Bendixen M, Jorgensen OD, Kronborg C (2016). Postoperative pain and quality of life after lobectomy via video-assisted thoracoscopic surgery or anterolateral thoracotomy for early stage lung cancer: a randomised controlled trial. Lancet Oncol.

[12] Wildgaard K, Ringsted TK, Hansen HJ (2016). Persistent postsurgical pain after video-assisted thoracic surgery--an observational study. Acta Anaesthesiol Scand.

[13] Gottschalk A, Ochroch EA (2008). Clinical and demographic characteristics of patients with chronic pain after major thoracotomy. Clin J Pain.

[14] Talbot RM, McCarthy KF, McCrory C (2015). Central and systemic inflammatory responses to thoracotomy -potential implications for acute and chronic postsurgical pain. J Neuroimmunol.

